# ADA2-Functionalized OMVs Remodel the Tumor Microenvironment in Pancreatic Cancer

**DOI:** 10.3390/pharmaceutics18080920

**Published:** 2026-07-27

**Authors:** Vahid Khalaj, Alyssa M. Waller, MacKenzie V. Demmel, Urvinder Kaur Sardarni, Jack T. Adams, Aidan Collier, Ana Maria Zaske, Majid Momeny, Jennifer M. Bailey-Lundberg, Ali Azhdarinia

**Affiliations:** 1 Institute of Molecular Medicine, The University of Texas Health Science Center at Houston, Houston, TX 77030, USA; vahid.khalaj@uth.tmc.edu (V.K.); majid.momeny@uth.tmc.edu (M.M.); 2 Department of Pathology, Microbiology and Immunology, The University of Nebraska Medical Center, Omaha, NE 68198, USA; alwaller@unmc.edu (A.M.W.); mdemmel@unmc.edu (M.V.D.); usardarni@unmc.edu (U.K.S.); 3 Fred & Pamela Buffett Cancer Center, The University of Nebraska Medical Center, Omaha, NE 68198, USA; 4 Department of Pediatric Surgery, McGovern Medical School, The University of Texas Health Science Center at Houston, Houston, TX 77030, USA; aidan.collier@uth.tmc.edu; 5 Department of Internal Medicine, McGovern Medical School, The University of Texas Health Science Center at Houston, Houston, TX 77030, USA; ana.m.zaske@uth.tmc.edu

**Keywords:** adenosine, adenosine deaminase 2, bacterial surface display, immunosuppression, outer membrane vesicle, PDAC, tumor microenvironment

## Abstract

**Background/Objectives:** Pancreatic ductal adenocarcinoma (PDAC) is a lethal malignancy with a desmoplastic, immunosuppressive tumor microenvironment enriched in extracellular adenosine. High adenosine levels disrupt anti-tumor immunity by engaging adenosine receptors on T cells, macrophages, and dendritic cells. In this context, strategies to reduce extracellular adenosine signaling within the TME are in focus. Among them, enzymatic degradation of extracellular adenosine levels has emerged as a promising approach to overcome immune suppression and restore anti-tumor immune responses. **Methods:** Here, we engineered an *E. coli* strain to express a surface-bound form of a bacterial ADA2 enzyme and used the outer membrane vesicles produced by this strain as nanoparticles carrying the functional enzyme. Our data indicate robust surface expression and enzymatic activity of ADA2 on OMV particles. **Results:** In a PDAC subcutaneous flank tumor model, intratumoral administration of OMV-ADA2 particles was associated with significantly increased infiltration of CD8^+^ T cells and Granzyme B expression and concomitant decreases in collagen deposition and α-smooth muscle actin (α-SMA) positive stromal cells, suggesting modulation of the desmoplastic stroma. **Conclusions:** These findings support further investigation of OMV–bADA2 as a potential platform to modulate tumor metabolism and stroma, activate anti-tumor immunity, and serve as a carrier for co-delivery of additional anticancer modalities in PDAC.

## 1. Introduction

Pancreatic ductal adenocarcinoma (PDAC) is the most common form of pancreatic cancer, originating from the exocrine compartment and accounting for more than 90% of all cases-. It is an exceptionally aggressive malignancy, typically diagnosed at advanced stages, with only a small fraction of ~2% detected early at stage 0 or I [1,2]. Standard treatment interventions such as surgery, chemotherapy, and radiation have provided limited improvements in long-term survival. Moreover, while immunotherapy has changed the treatment landscape of several malignancies, immune checkpoint inhibitors, including anti-CTLA-4, anti-PD-1, and anti-PD-L1 antibodies, have shown limited efficacy in this disease [1]. These limited responses are attributed to the genetic heterogeneity of the tumor and its uniquely immune suppressive tumor microenvironment (TME). The TME of PDAC is marked by dense desmoplastic stroma, aberrant vasculature, and significant domination by infiltrating myeloid cells such as CXCR2-expressing myeloid-derived suppressor cells (MDSCs), tumor-associated macrophages (TAMs), and dysfunctional, exhausted CD8^+^ T cells expressing multiple immune checkpoints like PD-1, LAG3, TIM3, and CTLA4 [3,4]. It has been shown that both cellular and acellular components of the TME actively promote tumor progression and therapy resistance [5]. As a result, introducing novel therapies targeting these components in combination with conventional treatments becomes essential.

A key and well-established metabolic contributor to immunosuppression in PDAC is the accumulation of extracellular adenosine [6]. High expression of membrane-bound ectonucleotidases CD39 and CD73 (NT5E) by tumor epithelial cells, infiltrating immune cells, and stromal cells results in rapid and stepwise conversion of ATP to AMP and subsequently to adenosine [7,8]. The elevated extracellular adenosine levels activate A2A and A2B receptors, which primarily mediate adenosine-induced immunosuppression [9,10,11,12,13,14]. Activation of A2A suppresses proximal T cell receptor (TCR) signaling and CD28 co-stimulation, as well as interleukin-2 receptor (IL-2R) signaling. This, in turn, attenuates T cell activation, proliferation, survival, and cytokine production. In addition, this pathway induces co-inhibitory receptors, including PD-1, CTLA4, LAG3, and TIM3, and drives CD4^+^ T cells toward a regulatory phenotype (Treg). Collectively, this adenosinergic axis promotes T cell exhaustion, expands Tregs, and polarizes macrophages toward tumor-promoting phenotypes [15,16]. In addition to A2A receptor signaling, activation of A2B receptors promotes MDSC expansion, impairs antitumor T cell responses, and enhances the production of angiogenic cytokines while suppressing macrophage inflammatory activity. These events further support an immunosuppressive tumor microenvironment [17,18].

Several strategies have been explored to disrupt adenosine-mediated immunosuppression, including inhibitory antibodies or small-molecules targeting the CD39-CD73 axis and adenosine receptors [11,13,19,20]. However, these approaches are often associated with significant systemic side effects, such as cardiovascular toxicity and immune-related adverse events, due to the broad physiological roles of adenosine signaling in the human body [16]. Moreover, genetic and metabolic redundancy in adenosine production pathways may hamper the long-term efficacy of such interventions. In light of these limitations, a strategy involving the direct enzymatic degradation of extracellular adenosine within the tumor microenvironment may provide more effective and localized immunometabolic reprogramming with reduced systemic toxicity.

Adenosine deaminase (ADA) is a critical enzyme in regulating adenosine signaling by unidirectional deamination of adenosine and deoxyadenosine to inosine and deoxyinosine, respectively. Two distinct ADA enzyme families have been characterized: ADA1 and the adenosine deaminase-related growth factors (ADGF/ADA2), also known as ADA2-like proteins [21,22]. ADA1 is found in both prokaryotic and eukaryotic cells as an intracellular monomer and acts to reduce intracellular adenosine concentrations [21]. In contrast, ADA2 is an extracellular dimeric enzyme optimally active at acidic pH (~6.5), with adenosine as its primary substrate [23,24]. ADA2 also shows a lower Km and higher catalytic activity at acidic pH, matching the conditions found in the hypoxic, adenosine-rich tumor microenvironment. Hence, it is particularly suited for targeted modulation of extracellular adenosine in tumors.

So far, limited efforts have focused on systemic administration of adenosine deaminase (ADA) to reduce adenosine-mediated immunosuppression in the tumor microenvironment. PEGylated human ADA2 exemplifies one such attempt to alleviate adenosine-driven immune suppression in tumors. Wang et al. showed that systemic PEG-ADA2 delivery reduced intratumoral adenosine, reprogrammed the tumor microenvironment toward immune activation, and inhibited tumor growth in mice [25]. This study also revealed that the higher levels of ADA2 were associated with improved survival in patients with increased levels of CD73 and CD39 expression in triple-negative breast cancer and non-small cell lung cancer. This correlation was not observed in patients with low levels of CD73 and CD39. These findings highlight the relevance of ADA2 and adenosine modulation in the TME. Similarly, PEGylated adenosine deaminase 1 (PEG-ADA), an FDA-approved enzyme replacement therapy for children with severe combined immunodeficiency (SCID), has been shown to relieve adenosine-mediated suppression of CD8^+^ T cells and enhance the efficacy of anti-PD-1 immunotherapy [26].

In a similar study, Yun and colleagues reported the successful engineering of ADA1-CAR T cells, enabling tumor microenvironment remodeling to favor antitumor immunity [27]. More recently, Li et al. reported the development of ADA/Ce6@tLipo, a multifunctional ultrasound-activated nanovesicle that integrates sonodynamic therapy, adenosine depletion via ADA, and immune checkpoint blockade [28]. Despite its promising efficacy, this approach faces several challenges, including a complex and labor-intensive fabrication process. Together, these issues highlight the importance of innovative strategies that facilitate ADA-mediated adenosine depletion within the complex tumor microenvironment and, at the same time, overcome the challenges and high costs associated with large-scale production of active human ADA or sophisticated synthetic nanoparticles.

Outer Membrane Vesicles (OMVs) are naturally occurring lipid bilayer vesicles extruded from the outer membrane of Gram-negative bacteria, containing outer membrane molecules and periplasmic components [29]. By selecting appropriate anchor proteins, recombinant proteins can be incorporated into OMVs without disrupting the vesicles or the growth of the parent bacterium. Diverse anchoring partners, such as autotransporters and outer membrane proteins, have been successfully used to achieve surface display of target proteins while maintaining the nanosized structure essential for efficient drug delivery [30,31]. As a result, OMVs have emerged as promising nanoparticle delivery platforms for vaccine development and cancer therapy [31,32,33]. Bacterial outer membrane vesicles present several advantages, including tumor-homing capability through the enhanced permeability and retention (EPR) effect, innate immunomodulatory properties, and the capacity for stable display of functional proteins on their surface [34,35]. Furthermore, OMVs are cost-effective due to the inexpensive cultivation of bacteria that generate these vesicles and have shown an acceptable safety profile, particularly in OMV-based vaccine formulations [35,36]. They can also encapsulate a variety of therapeutic agents, including chemotherapeutics, as well as recombinant peptides with diverse anti-tumor activity, which can be produced directly by genetically engineered bacteria. This capability further enhances their potential as multifunctional drug delivery systems [37,38,39].

**Figure 1:**
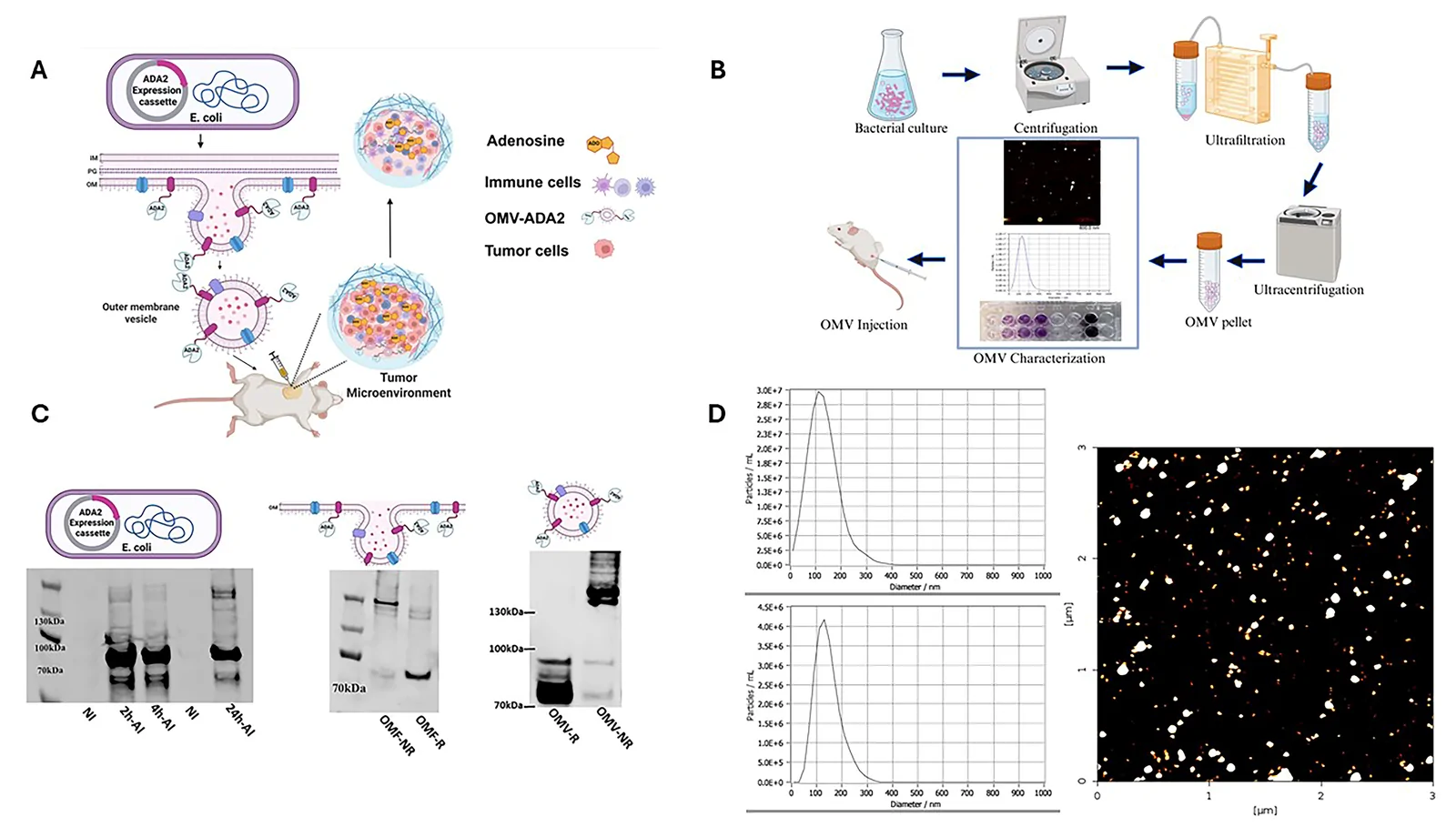
Preparation, characterization, and tumoral administration of *E. coli*-generated OMV-ADA2 particles. (**A**) Schematic of the OMV-bADA2 adenosine depletion strategy in the tumor microenvironment. We hypothesize that ADA2-mediated adenosine degradation leads to adenosine depletion and subsequent activation of immune cells in the TME. (**B**) Workflow for OMV isolation, purification, characterization, and intratumoral administration. (**C**) Western blot analysis confirming ADA2 expression in engineered *E. coli* and its incorporation into OMVs. (Right panel) total cell lysate; NI, non-induced; AI, after induction. (Middle panel) outer membrane fraction; NR, non-reduced condition; R, reduced condition. (Left panel) outer membrane vesicles; NR, non-reduced condition; R, reduced condition. Blots were probed with anti-His antibody. (**D**) Nanoparticle tracking analysis of naïve OMVs (**top**) and OMV-bADA2 particles (**bottom**) showing size distributions, and AFM image of OMVs.

Building on these unique features, we hypothesized that OMVs carrying an active form of ADA can effectively modulate the tumor microenvironment. Recently, the bacterial homologue of adenosine deaminase 2 (bADA2) has been identified, exhibiting 30–32% sequence identity with human ADA2, including a conserved dimerization domain [40]. More importantly, bacterial and eukaryotic ADA2 share a very similar catalytic site, with 13 of 16 amino acids identical in bacterial and human ADA2. Biochemical characterization revealed that the bADA2 dimer is stabilized by an intermolecular disulfide bond formed by Cys190 residues. Interestingly, the recombinant bADA2 demonstrated an impressive affinity (Km) of 96.44 ± 66.82 µM, which is closer to that of the highly active invertebrate ADGF (50 µM) than the comparatively less active human ADA2 (2.53 mM). The optimal activity of bADA2 was observed at pH 6.8, which closely corresponds to the pH optimum of human ADA2. These findings suggest that the bacterial ADA2 could be a potent and cost-effective enzymatic substitute to reduce extracellular adenosine levels. In this proof-of-concept study, we report the development and biological characterization of engineered OMVs displaying bADA2 on their surface as a novel and promising tool for depletion of adenosine, reversion of immunosuppression, and reactivation of antitumor immunity (Figure 1A).

## 2. Methods

### 2.1. Bacterial Strains, Cell Lines, and Reagents

The *E. coli* strains utilized in this study were DH5α (NEB^®^ 5-alpha Competent *E. coli*, New England Biolabs, Ipswich, MA, USA) for plasmid propagation and ClearColi^®^ BL21(DE3) (Biosearch Technologies, Radnor, PA, USA) for the surface expression of bADA2 and the production of engineered OMVs. ClearColi^®^, an LPS mutant strain of *E. coli*, generates a modified LPS structure, which significantly reduces endotoxicity and eliminates immunostimulant membrane antigens, making it particularly advantageous for OMV production. *E. coli* strains were grown in Luria–Bertani (LB) medium [1% (*w*/*v*) tryptone, 0.5% (*w*/*v*) yeast extract, and 1% (*w*/*v*) NaCl, pH 7.0]. The growth medium was supplemented with Kanamycin (50 μg/mL) when needed. KPC cells were cultured in DMEM supplemented with 10% fetal bovine serum (FBS), 100 U/mL penicillin, and 100 μg/mL streptomycin (Thermo Fisher Scientific, Waltham, MA, USA) at 37 °C in a humidified atmosphere with 5% CO_2_.

### 2.2. Construction of E. coli Strain Expressing Surface-Displayed Recombinant Bacterial ADA2

The reported 502 amino acid sequence of *Elizabethkingia anophelis* adenosine deaminase 2 (WP_021346688.1) was analyzed for a signal sequence. Amino acids 1–24 were annotated as signal sequence using an online prediction tool (https://services.healthtech.dtu.dk/services/SignalP-4.1/ (accessed on 3 March 2023)). In the engineered bADA2 expression cassette, the putative signal sequence was excised, and the truncated sequence was subsequently linked to the carboxy-terminal region of a bacterial surface anchoring sequence, Lpp’OmpA, using a G4S linker. Lpp’OmpA is a chimera created by Georgiou and colleagues [41,42]. It comprises the signal peptide and the initial nine residues of Braun’s lipoprotein, also known as Lpp (Lpp’), which are responsible for targeting to the outer membranePl. This portion is fused with five of the eight membrane-spanning segments of the OmpA porin, spanning residues 46–159. OmpA is a prevalent, monomeric outer membrane protein with a transmembrane domain consisting of an eight-stranded β-barrel. The Lpp domain targets and anchors the fused protein to the outer membrane, while the OmpA domain is essential for the surface expression of the passenger protein [43]. To finalize the Lpp’OmpA-bADA2 construct, a 6X His tag sequence was added to the C-terminal of the designed fusion polypeptide. The final sequence (632 amino acids) was reverse-translated into a DNA sequence using an online tool (https://www.geneinfinity.org/sms/sms_backtranslation.html (accessed on 3 March 2023)) based on *Escherichia coli* K12 codon usage. Flanking restriction sites, *Nde*I and *Bam*HI, were incorporated at the gene termini. The resulting sequence was then provided to Biomatik (Kitchener, ON, Canada) for whole gene synthesis and cloning into the *Nde*I/*Bam*HI site of the plasmid vector pET-26b (Novagen Corporation, Madison, WI, USA).

### 2.3. Expression Analysis of Recombinant Lpp’OmpA-bADA2

The electrocompetent ClearColi^®^ BL21(DE3) bacteria were transformed with the pET-26b-Lpp’OmpA-bADA2 expression construct (hereinafter referred to as bADA2-Display). A single colony was selected and cultured. Protein expression was induced by adding isopropyl β-D-1-thiogalactopyranoside (IPTG) to a final concentration of 1 mM when the culture reached an OD600 of ~0.6. The expression of the recombinant bADA2-Display construct was confirmed using SDS-PAGE and Western blot analysis following standard protocols. Total bacterial cell lysates were prepared using GoldBio bacterial lysis buffer according to the manufacturer’s instructions (Gold Biotechnology, St. Louis, MO, USA). The clear lysate supernatant was collected and used for SDS-PAGE analysis.

For Western blotting, total bacterial lysates were separated using SurePAGE™ Precast Gels (GenScript, Piscataway, NJ, USA) and transferred to a nitrocellulose membrane. The membranes were blocked with Intercept Blocking Buffer (LI-COR) and incubated with specific primary antibody, followed by IR-labeled secondary antibodies. Immunoreactive bands were visualized using the LI-COR Odyssey Western blot imager (LI-COR Biosciences, Lincoln, NE, USA).

To detect the recombinant bADA2-Display in the outer membrane, the outer membrane fraction was isolated following the protocol described by Park et al. [44]. Harvested *E. coli* cells were resuspended in 0.2 M Tris–HCl buffer (pH 8.0) and lysed using lysozyme (200 μg/mL final concentration) in the presence of 20 mM sucrose, 0.2 mM EDTA, and protease inhibitor cocktail for 10 min at room temperature. Outer membrane proteins were extracted by adding an equal volume of extraction buffer (2% Triton X-100, 50 mM Tris–HCl, 10 mM MgCl_2_) containing DNAse (10 μg/mL). The mixture was incubated on ice for 30 min and centrifuged at 4000 rpm for 5 min to remove debris. The outer membrane-containing supernatant was centrifuged at 18,000 rpm for 10 min, and the resulting pellet was washed twice with PBS and dissolved in PBS for SDS-PAGE, Western blotting, and the enzyme activity assay.

### 2.4. ADA Enzymatic Activity Assay

The ADA activity of the outer membrane fraction and outer membrane vesicles was measured using a commercial colorimetric kit (E-BC-K197-M, Elabscience, Houston, TX, USA). The assay is based on ADA-catalyzed hydrolysis of adenosine to inosine, followed by conversion to hypoxanthine by purine riboside phosphorylase at 37 °C. The hypoxanthine then reacts with xanthine oxidase to produce hydrogen peroxide. In the presence of peroxidase, 4-aminopyrine, and a chromogenic substrate, a purple-colored product is formed. The absorbance at 550 nm is directly proportional to ADA activity.

In brief, 10 μL of standard solutions or sample (outer membrane fraction or OMVs) were added to designated wells, followed by 180 μL of working solution and 90 μL of chromogenic agent. The plate was incubated at 37 °C for seven minutes, and the OD at 550 nm was measured (A1). The incubation then continued for 10 min at 37 °C and was read as A2. Activity quantification was performed using a standard curve (y = ax + b) and calculated according to the formula below: ADA activity (U/L) = (A2 − A1 − b) ÷ a × 1000* ÷ T × f.

Where A1: The OD value after the first incubation for 7 min; A2: The OD value after the second incubation for 10 min; T: The second incubation time, 10 min; 1000*: 1 mmol = 1000 μmol; f: dilution factor. To assess the adenosine-depleting capacity of OMV-bADA2 particles, adenosine was added to serum-free medium to a final concentration of 200 µM and incubated at room temperature with 2.5 µL (OMV-bADA2 per tube, with separate tubes prepared for each time point. Reactions were stopped at 10, 20, 30, 60, and 120 min by rapid freezing on dry ice, followed by storage at −80 °C, and the samples were subsequently analyzed for adenosine by liquid chromatography–mass spectrometry (LC-MS). Experimental conditions included OMV-bADA2 at all time points, naïve OMVs at 30, 60, and 120 min, and a no-OMV control at 120 min, with all samples prepared in duplicate.

To test the stability of OMV-bADA2-associated enzyme activity, a stability assay was performed. OMV-bADA2 particles were diluted in either PBS or 50% (*v*/*v*) mouse serum to achieve a final enzyme activity of 4 U/L in a total volume of 200 μL. Duplicate aliquots were collected at 0, 2, 4, 8, and 24 h and immediately stored at −80 °C until analysis. ADA activity was quantified using the above-mentioned method, and results were reported as activity (U/L) at each time point.

### 2.5. Preparation of OMV-bADA2-Display

For small-scale preparation, OMVs were isolated from *E. coli* ClearColi^®^ BL21(DE3) using the ExoBacteria™ OMV Isolation Kit (System Biosciences, Newark, CA, USA) according to the manufacturer’s protocol. Briefly, bADA2-Display expression was induced in a 30 mL culture volume, as described above. Afterward, cells were harvested by centrifugation at 5000× *g* for 20 min, and the cell-free supernatant was sequentially filtered through 0.45 µm and 0.2 µm filters. The filtrate was then subjected to column-based purification using the OMV isolation kit. The purified OMVs were stored at −80 °C until use. The total protein content of the OMVs was quantified using the Bradford assay, with bovine serum albumin as the standard.

For large-scale preparations, we followed a previously published method [45]. In brief, *E. coli* cultures were grown overnight and used to inoculate 2 L of LB medium. Cultures were grown with shaking at 37 °C and, as described above, recombinant protein expression was induced by IPTG. After cultivation, bacterial cells were removed by centrifugation at 5000× *g* for 20 min. The cell-free supernatant was sequentially filtered through 0.45 μm and 0.2 μm filters and concentrated to 150 mL using a 100 kDa cutoff Vivaflow^®^ 200 Tangential Flow Filtration Cassette (Sartorius, Bohemia, NY, USA). OMVs were then collected by ultracentrifugation at 45,000 rpm (45Ti rotor, Beckman-Coulter, Avanti™ J-20 XPI, Brea, CA, USA) for 2 h at 4 °C, washed once with PBS by repeating ultracentrifugation, and finally resuspended in 2 mL PBS and kept at −80 °C (Figure 1B).

### 2.6. Nanoparticle Characterization via ZetaView Z-NTA

The size, concentration, and distribution of OMV nanoparticles were analyzed using Nanoparticle Tracking Analysis (NTA) on a ZetaView^®^ Z-NTA instrument (Particle Metrix, Holly Springs, NC, USA). OMV samples were equilibrated to room temperature and diluted 1:2000 in PBS. A 1 mL aliquot of the diluted sample was introduced into the instrument. Particle size distribution, concentration, and zeta potential measurements were performed using ZetaView software (version 8.06.01). The analysis utilized a laser wavelength of 488 nm with scatter detection, a sensitivity setting of 58.0, a shutter setting of 100, and a frame rate of 30 frames per second with high video resolution. The sample temperature was measured at 22.59 °C, with a pH of 7.0 and a conductivity of 10,172.00 µS/cm.

### 2.7. AFM Analysis of OMV-bADA2 Particles

Freshly cleaved mica surface (highest grade V1 mica discs 12 mm, Ted Pella, Inc., Redding, CA, USA.) was treated with 10 µL of 3-Aminopropyltriethoxysilane (APTES:1 µM in miliQ-water) for 5 min and rinsed with 2 mL of miliQ-water. A drop of 10 µL of the OMV-ADA2 suspension (50 µg/mL) was incubated for 5 min on the functionalized mica (AP-mica), rinsed with 100 µL of miliQ-water, fast dried by suction and scanned immediately after preparation.

Atomic Force Microscopy (AFM) was conducted at the UTHealth—AFM Core Facility using the JPK NanoWizard V AFM (Bruker Nano, Inc., Santa Barbara, CA, USA).

This system is integrated into a Nikon TE2000-E inverted optical microscope (Nikon Instruments Inc., Melville, NY, USA) to facilitate bright field imaging. AFM images were processed using the JPK DataProcessing (DP) Software version 8.1.69 (copyright Bruker Nano). High resolution images of OMVs were obtained using TESPA cantilevers (*fo* = 301–345 kHz, *k* = 20–80 N/m, Bruker Corporation, Santa Barbara, CA, USA). The particle’s topography was determined using Peak Force Tapping mode operated in air at a velocity of 2.4 µm/s. Scan sizes ranged from 3.0 to 1.5 µm.

### 2.8. Animal Model

All mouse model procedures followed UNMC’s Institutional Animal Care and Use Committee (IACUC) protocol #24-047-09-FC, approval date 11 September 2024, and adhere to ARRIVE guidelines. KPC cells were derived in the Tuveson Lab from *Kras^LSL-G12D/+^*; *Trp53^LSL-R172H/+^*; *Pdx1-Cre* mice, which develop PDAC. 1 × 10^6^ KPC cells were prepared in PBS:Matrigel mix (1:1) and injected subcutaneously into the right flank of 8–10-week-old male C57BL/6 mice. Tumor size was measured once per week with vernier calipers.

### 2.9. OMV-bADA2 Treatment

Treatment with OMV-bADA2 began on day 14 post KPC cell injection, when tumors reached 200–500 mm^3^. For this study, treatment groups included untreated control, 5 μg OMV-naïve (diluted in PBS), 0.5 μg OMV-bADA2 (corresponding to 4.8 × 10^7^ particles, diluted in PBS), and 5 μg OMV-bADA2 (corresponding to 4.8 × 10^8^ particles, diluted in PBS), with *n* = 5 animals per group. In all cases, OMV doses (0.5 or 5 μg) refer to the total OMV protein mass. Mice were housed with *n* = 5 mice per cage. Mice were injected intratumorally on days 14, 15, and 16, for a total of three injections. On day 16, two mice per group were euthanized by isoflurane overdose two hours after treatment, and tumor tissues were collected. On day 17, the remaining three mice per group were euthanized 24 h after the last treatment, and tumor tissues were collected.

### 2.10. Immunohistochemistry and ImageJ Analysis

Tissues were fixed in zinc-buffered formalin, processed, and embedded in paraffin. Sections were placed on positively charged slides and baked at 60 °C for 30 min. Slides were deparaffinized in Histo-clear and gradually rehydrated in graded ethanol to PBS. Heat-mediated antigen retrieval was performed using a microwave-based method and a pH 6 solution (Vector Laboratories, Newark, CA, USA, H-3300). Sections were blocked for 1 h at room temperature (RT) in 10% FBS in PBST. The primary antibody αSMA (1:1000, ab5694, Abcam, Waltham, MA, USA) was diluted in a blocking solution, added to tissue sections, and incubated overnight at 4 °C. Tissues were then washed three times with PBS for 5 min and incubated in secondary antibody (Vector Laboratories, BA-1000-1.5) at RT for 30 min at a 1:500 dilution in blocking buffer. Next, tissues were washed three times with PBS for 5 min and incubated using the Vectastain ABC kit Peroxidase Standard (Vector Laboratories, PK4000). Tissues were washed three times with PBS for 5 min, then the DAB peroxidase (HRP) Substrate kit (Vector Laboratories, SK-4100) was used. Tissues were rinsed in tap water for 2 changes of 5 min each, then stained with hematoxylin, and gradually dehydrated in graded ethanol. Tissues were cleared in Histo-clear and mounted. Analysis of immunohistochemistry images was performed using ImageJ software Version 1.54g (https://imagej.net; accessed on 22 May 2025). Five to ten representative fields per tissue were used, depending on tissue size. The color threshold tool was used to determine positive staining in each field.

### 2.11. Hematoxylin and Eosin Staining

Formalin-fixed, paraffin-embedded tissues were sectioned and mounted on charged glass slides. Slides were placed in histoclear for 8 min, 100% ethanol for 4 min, and 95% for 2 min. Slides were put in the following order for 3 min each: tap water, hematoxylin, and tap water. Next, tissues were left in the following order for 1 min each: clarifier, tap water, bluing agent, water, 95% ethanol, eosin, and 95% ethanol. Slides were placed for 4 min in 100% ethanol and 4 min in Histo-clear, then mounted.

### 2.12. Immunohistochemistry Protocol for CD8α and Granzyme B

Tissues were fixed in zinc-buffered formalin, processed, and embedded in paraffin. Sections were placed on positively charged slides and baked at 60 °C for 30 min. Slides were deparaffinized in Histo-clear and gradually rehydrated in graded ethanol to PBS. Heat-mediated antigen retrieval was performed using a microwave-based method and either a citrate buffer (pH 6.0, Abcam, AB93678) or Tris-EDTA buffer (pH 9.0, Abcam, AB93684). Sections were blocked for 1 h at room temperature (RT) in 10% FBS in PBST. The primary antibodies αSMA (1:1000, ab5694, Abcam), CD8α (1:250, ab217344, Abcam), and granzyme B (1:200, ab255598, Abcam) were diluted in a blocking solution, added to tissue sections, and incubated overnight at 4 °C. Tissues were then washed three times with PBS for 5 min and incubated in secondary antibody (Vector Laboratories, BA-1000-1.5) at RT for 30 min at a 1:500 dilution in blocking buffer. Next, tissues were washed three times with PBS for 5 min and incubated using the Vectastain ABC kit Peroxidase Standard (Vector Laboratories, PK4000). Tissues were washed three times with PBS for 5 min, then the DAB peroxidase (HRP) Substrate kit (Vector Laboratories, SK-4100) was used. Tissues were rinsed in tap water for 2 changes of 5 min each, then counterstained with hematoxylin, and gradually dehydrated in graded ethanol. Tissues were cleared in Histo-clear and mounted. Analysis of immunohistochemistry images was performed using ImageJ 1.54g software (https://imagej.net, accessed on 16 July 2026). Five to ten representative fields per tissue were used, depending on tissue size. The color threshold tool was used to determine positive staining in each field.

### 2.13. Trichrome Staining

Slides were stained according to the manufacturer’s protocol for the trichrome staining kit (Abcam, ab150686). They were first deparaffinized for 9 min in Histo-clear and gradually rehydrated through graded ethanol to distilled water, then incubated in preheated Bouin’s fluid for 60 min and rinsed in running tap water until the sections appeared clear. After a 4 min rinse in tap water, slides were stained with working Weigert’s iron hematoxylin for 5 min, rinsed for 2 min in running tap water, and then stained with Biebrich scarlet/acid fuchsin solution for 15 min. Following a rinse in distilled water, slides were incubated in phosphomolybdic/phosphotungstic acid solution for 10 min and, without further rinsing, stained with aniline blue solution for 10 min. They were then rinsed in distilled water, immersed in acetic acid solution for 5 min, dehydrated in 95% and 100% ethanol, cleared in Histo-clear, and mounted.

### 2.14. Flow Cytometry

Tumors were dissociated immediately after extraction. They were minced, digested in 1 mL collagenase (Worthington, Lakewood, NJ, USA, LS004186), and placed in a shaker for 30 min at 37 °C, 200 rpm. The mixture was passed through a 30 μm filter and rinsed with 0.04% BSA/PBS. Cells were centrifuged at 453× *g*, 4 °C for 5 min. Isolated cells were washed with PBS and incubated with Live/Dead Fix Blue (Thermo Fisher Scientific, L34962). Samples were centrifuged, red blood cells were lysed, and cells were fixed by incubation with 1X RBC Lysis/Fixation Solution (BioLegend, San Diego, CA, USA, 442401) for 15 min. Samples were then centrifuged, resuspended in 1 mL of PBS, and stored overnight at 4 °C. The next day, samples were incubated with Fc Receptor Block (BD Biosciences, Milpitas, CA, USA, 553142) for 10 min to limit non-specific antibody binding. The samples were then incubated for 30 min with 150 μL of an antibody cocktail containing CD3 (BD Biosciences, 563565, 1:30, RRID: AB_2738278), CD163 (Thermo Fisher Scientific, 367163182, 1:120, RRID: AB_3074029), CD274 (BioLegend, 124315, 1:30, RRID: AB_10897097), CD11c (BioLegend, 117331, 1:30, RRID: AB_10900261), CD86 (BioLegend, 105035, 1:30, RRID: AB_11126147), CD80 (BD Biosciens, 740888, 1:30, RRID: AB_2740537), CD4 (BioLegend, 100406, 1:300, RRID: AB_312691), Ly-6G (BD Biosciences, 566435, 1:30, RRID: AB_2739730), CD38 (BioLegend, 102712, 1:120, RRID: AB_312933), CD45 (BioLegend, 103128, 1:300, RRID: AB_493715), CD8a (BioLegend, 100714, 1:30, RRID: AB_312753), CD279 (BioLegend, 135205, 1:30, RRID: AB_1877232), CD161 (BioLegend, 108748, 1:120, RRID: AB_2564219), and MHC II (BioLegend, 107630, 1:500, RRID: AB_2069376). An antibody cocktail was made in 66% 0.5% BSA/PBS and 33% Brilliant Stain Buffer (BD Biosciences, 563794) to prevent interactions between the fluorescent dyes and staining artifacts caused by the multiple Brilliant dyes used. Samples were centrifuged and resuspended in 0.5% BSA/PBS. Data was acquired using a BD LSRFortessa and analyzed with FlowJo v10.10.0.

## 3. Results

### 3.1. Validation and Analysis of Recombinant bADA2-Display

To validate the expression of the bADA2-Display fusion construct, a single colony of recombinant ClearColi^®^ BL21(DE3) was cultured and induced with IPTG. Western blot analysis of total lysate using anti-6xHis antibody (1:5000 dilution, Proteintech, Rosemont, IL, USA) confirmed successful expression of the target protein by the presence of both dimeric and monomeric forms of surface-displayed bADA2 under non-reducing and reducing conditions, respectively. The theoretical molecular weights of the monomer and dimer are approximately 72 kDa and 145 kDa, respectively, consistent with the migration pattern observed in SDS-PAGE (Figure 1C, left panel). Furthermore, immunoblotting with the same antibody confirmed the presence of the fusion protein in the isolated outer membrane fraction, indicating proper membrane localization (Figure 1C, middle panel).

### 3.2. Isolation and Characterization of OMV-bADA2 Particles

Bacterial OMV-bADA2 particles were isolated as described above. In a large-scale preparation, particle size analysis using ZetaView^®^ Z-NTA showed a median diameter of 126.2 nm and a mean diameter of 143.2 nm, with a particle concentration of 4.8 × 10^10^ particles/mL, which represents only a modest increase compared with naïve OMVs (median 114.4 nm; mean 131.1 nm) and remains well within the typical OMV size range (Figure 1D). To confirm OMV integrity and the presence of bADA2-Display, SDS-PAGE and Western blotting were performed. Since outer membrane vesicles are rich in outer membrane proteins (OMPs), SDS-PAGE revealed a major band at ~37 kDa, consistent with typical OMPs, indicating successful OMV isolation (Supplementary Figure S1C) [46]. Immunoblotting using anti-6xHis antibody detected both monomeric and dimeric forms of bADA2 in OMVs (Figure 1C, right panel).

### 3.3. Adenosine Deaminase Activity of OMV-bADA2 Particles

**Figure 2:**
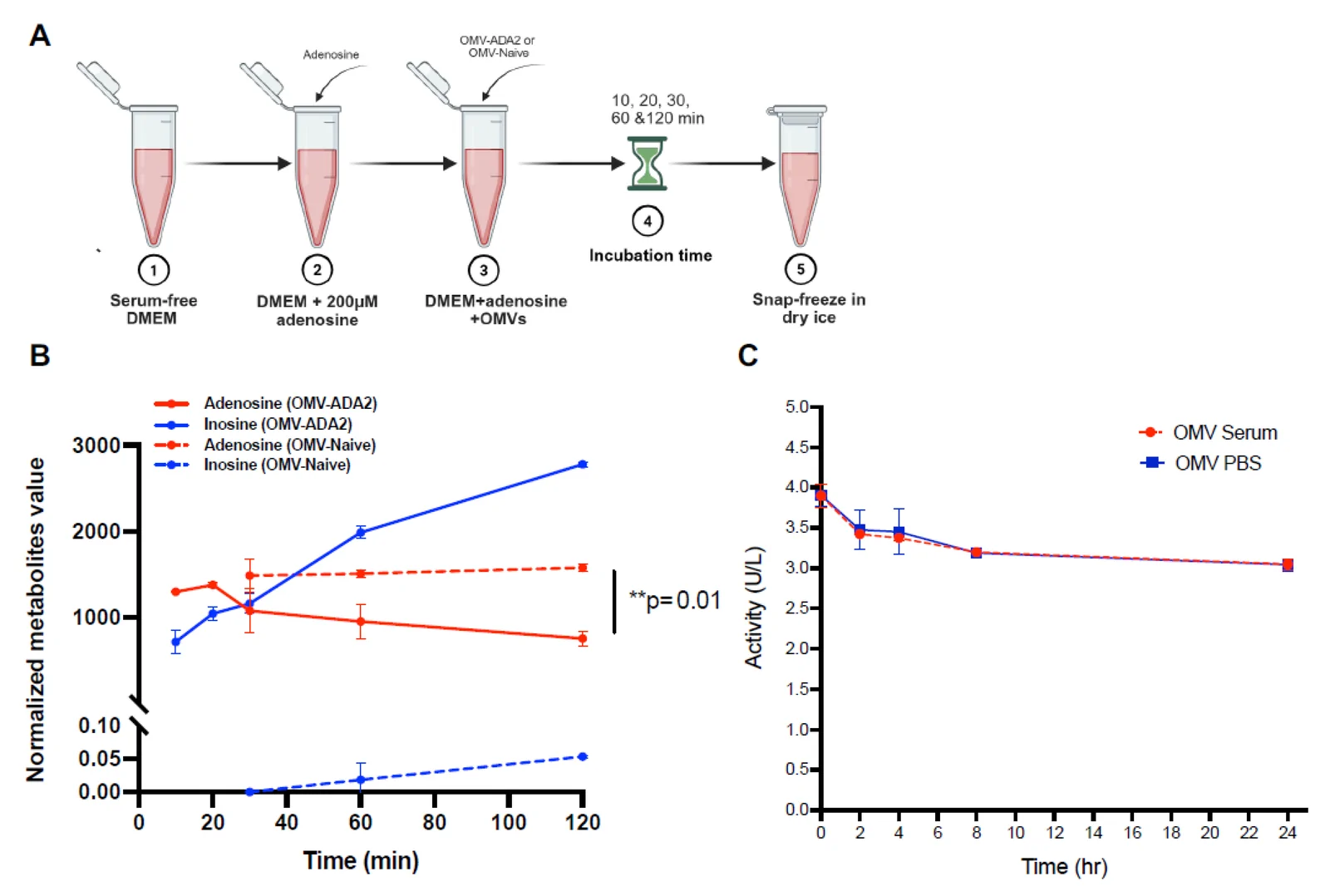
OMV-bADA2 reduces adenosine levels and retains enzymatic activity in vitro. (**A**) Schematic of in vitro experiment to monitor the activity of OMV-naïve compared to OMV-ADA2. (**B**) OMV-ADA2 significantly decreases the concentration of adenosine in DMEM over a time frame of 2 h (** *p* = 0.01). In the same time frame, OMV-naïve does not reduce adenosine concentration in DMEM. (**C**) The adenosine deaminase activity associated with OMV-bADA2 particles remained stable over 24 h in both mouse serum and PBS. Data are presented as mean ± SD.

The purified outer membrane fraction and isolated OMVs from the recombinant bacteria expressing bADA2-Display were subjected to ADA activity assay using the commercial colorimetric kit described above. Both samples showed strong adenosine deaminase activity. Because the kit’s detection range was 0.03–99 U/L, the samples were diluted up to 500 times. The adenosine deaminase activity of the outer membrane fraction was approximately 300 U/L. For isolated OMVs, the activity was calculated as 250 U/L (500 U/gram total OMV protein). These values represent a prototype assay using a typical large-scale preparation of OMVs and outer membrane fraction. In an adenosine depletion assay using serum-free medium, OMV-bADA2 treatment led to a time-dependent decrease in adenosine signal, accompanied by a mutual increase in inosine, whereas naïve OMVs and the no-OMV control showed minimal change over the same interval (Figure 2B). The adenosine deaminase activity associated with OMV-bADA2 demonstrated substantial stability in both PBS and 50% mouse serum. The time-course profiles in serum and PBS were nearly identical, indicating that the OMV formulation effectively preserves bADA2 enzymatic function under both buffer and physiologically relevant conditions (Figure 2C).

### 3.4. Immunomodulation and Stromal Remodeling Induced by OMV-bADA2

**Figure 3:**
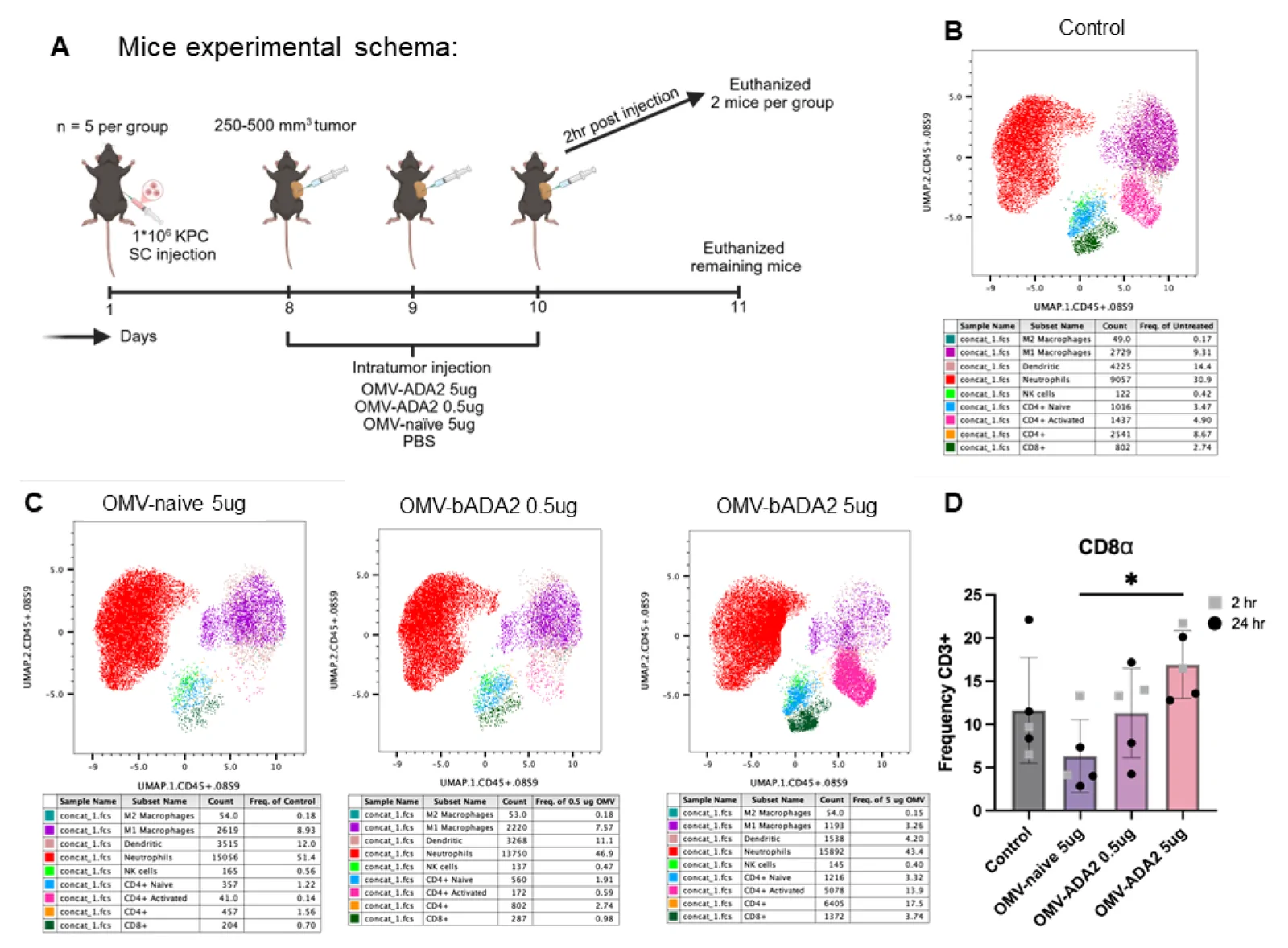
OMV-bADA2 intratumoral delivery significantly increases the % of CD8α+ T cells in KPC subcutaneous flank tumors. (**A**) Schematic of the preclinical model to evaluate effects of OMV-bADA2 compared to OMV-naïve on the tumor immune microenvironment. (**B**) UMAP of flow data in a control KPC flank tumor. (**C**) UMAPs of OMV-Naïve, OMV-bADA2 (0.5 µg dose) and OMV-bADA2 (5.0 µg dose) show significant changes in the percentage of CD8α+ T cells (* *p* < 0.05), quantified in (**D**). A one-way ANOVA using GraphPad Prism 10.6.1 was used for the statistical test.

To examine whether depletion of adenosine in the TME through OMV-bADA2 treatment reshapes tumor-infiltrating immune cells, tumor samples were stained with a panel of myeloid and T-cell markers and subjected to high-dimensional flow cytometry/UMAP clustering. Compared with OMV-naïve tumors, OMV-bADA2, particularly at the 5 µg (2.5 mU activity) dose, was associated with an increase in activated CD8^+^ T cells compared to the OMV-naïve treated tumors (Figure 3).

**Figure 4:**
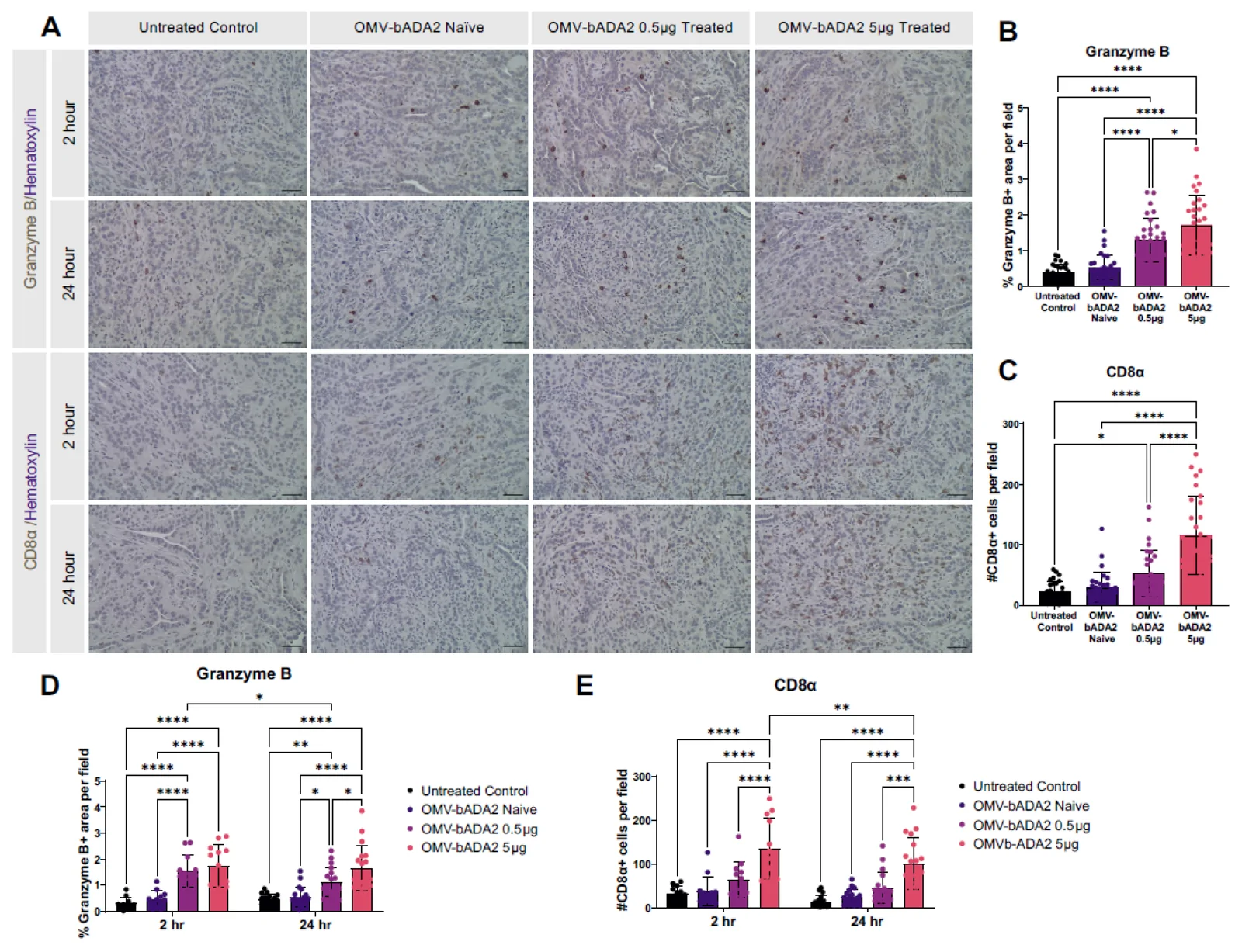
Treatment with OMV-bADA2 induces Granzyme B expression and infiltration of CD8^+^ T cells in KPC subcutaneous tumors. (**A**) Representative IHC images of Granzyme B and CD8α in KPC subcutaneous tumor tissue from untreated control, OMV-bADA2 naïve, OMV-bADA2 (0.5 μg), and OMV-bADA2 (5 μg) treated WT mice. Animals quantified per group: 2–5. Panels analyzed per mouse: 5–10. Data is presented as Mean ± SD. Images taken at 20×, scale bars represent 50 µm. (**B**) Composite quantification of percent granzyme b+ area per field across 2 h and 24 h timepoints. Treatment with 5 μg OMV-bADA2 significantly increased the Granzyme B+ area when compared to the untreated control, the OMV-bADA2 naïve treated tumors, and the OMV-bADA2 0.5 μg treated tumors. Treatment with 0.5 μg OMV-bADA2 significantly increased granzyme B+ area when compared to the untreated control and the OMV-bADA2 naïve treated tumors. (**C**) Composite quantification of CD8α+ cells per field at 2 h and 24 h timepoints. Treatment with 5 μg OMV-bADA2 significantly increased the number of CD8α + cells per field when compared to the 0.5 μg OMV-bADA2 treated tumors, the OMV-bADA2 naïve treated tumors, and the untreated control. Treatment with 0.5 μg OMV-bADA2 significantly increased the number of CD8α+ cells per field when compared to the untreated control. (**D**) Timepoint-stratified quantification comparing granzyme B+ area at 2 h and 24 h. (**E**) Timepoint-stratified quantification comparing CD8α+ cells per field at 2 h and 24 h. Composite timepoint comparisons were performed using one-way ANOVA and timepoint-stratified comparisons were performed using two-way ANOVA. * *p* ≤ 0.05; ** *p* ≤ 0.01; *** *p* ≤ 0.001; **** *p* ≤ 0.0001.

To evaluate CD8α and Granzyme B infiltration into the tumors, we used immunohistochemistry to stain for both markers. These data show at both 2 and 24 h after the final OMV injections, there is a significant increase in both Granzyme B and CD8α staining in the OMV-bADA2 5 µg treated tumors compared to the untreated controls, OMV-naïve or OMV-bADA2 0.5 ug treated tumors (Figure 4). These data show that the OMV-bADA2 significantly increased the infiltration of cytotoxic CD8^+^ T cells into KPC tumors.

**Figure 5:**
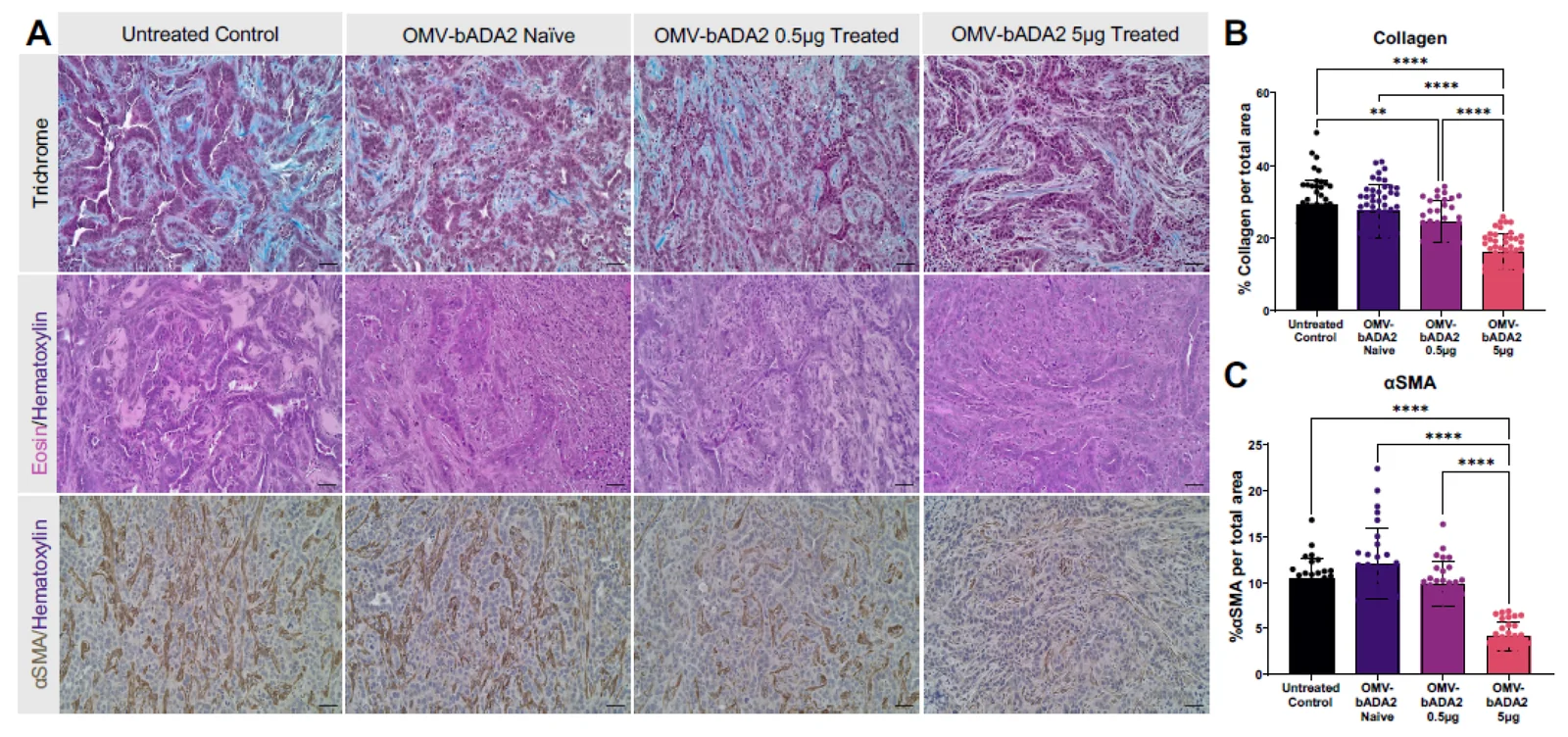
Treatment with OMV-bADA2 reduces collagen deposition and αSMA+ area in KPC subcutaneous tumors. (**A**) Representative images of trichrome, hematoxylin and eosin, and IHC staining of KPC subcutaneous tumor tissue from untreated control, OMV-bADA2 naïve, OMV-bADA2 (0.5 μg), and OMV-bADA2 (5 μg) treated WT mice. Animals quantified per group: 5. Panels analyzed per mouse: 5–10. Data is presented as Mean ± SD. Images taken at 20× scale bars represent 50 uM. (**B**) Quantification of collagen deposition (trichrome + area). Treatment with 5 μg OMV-bADA2 significantly reduced collagen deposition when compared to the untreated control, the OMV-bADA2 naïve treated tumors, and the OMV-bADA2 0.5 μg treated tumors. Treatment with 0.5 μg OMV-bADA2 significantly reduced collagen deposition when compared to the untreated control. (**C**) Quantification of αSMA+ area. Treatment with 5 μg OMV-bADA2 significantly reduced the percentage of αSMA+ area per field when compared to the 0.5 μg OMV-bADA2 treated tumors, the OMV-bADA2 naïve treated tumors, and the untreated control. Data was analyzed by one-way ANOVA. ** *p* ≤ 0.01; **** *p* ≤ 0.0001.

We next quantified collagen deposition by Masson’s trichrome staining and assessed αSMA-positive stromal cells by immunohistochemistry. Trichrome staining showed abundant collagen deposition in tumors from untreated and OMV-naïve groups, whereas both low-dose (0.5 µg/0.25 mU activity) and high-dose (5 µg/2.5 mU activity) OMV-bADA2 significantly decreased trichrome-positive collagen area, with the greatest reduction observed at 5 µg (Figure 5). Consistently, αSMA Immunohistochemistry revealed a dense population of α-SMA^+^ stromal cells in control and OMV-naïve tumors, whereas OMV-bADA2 treatment produced a significant reduction in the percentage of α-SMA^+^ area per field, most prominently at the 5 µg dose. Based on these histologic and quantitative analyses, OMV-bADA2 treatment reduced tumor fibrosis in a dose-dependent manner.

## 4. Discussion

High extracellular adenosine is a key metabolic driver of immune suppression in pancreatic ductal adenocarcinoma (PDAC) and a major contributor to the failure of immunotherapy in this cancer. Various strategies have been implemented to block adenosine production (via CD39/CD73 inhibitors) or signaling (via A2A/A2B antagonists); however, these approaches are often limited by systemic toxicity and metabolic redundancy [16,47,48]. Direct enzymatic degradation of extracellular adenosine using recombinant and PEGylated ADA enzymes has shown promise in reversing adenosine-driven immune suppression, but large-scale production and delivery remain challenging [25,26,49].

The present study builds on prior work characterizing the adenosine deaminase activity of a bacterial ADA2 (bADA2) from *Elizabethkingia anophelis* and explores its potential application when displayed on outer membrane vesicles. This bacterial enzyme is identified as an evolutionary precursor of eukaryotic ADA2 and shares conserved active site and dimerization motifs that are important for its function [40]. By engineering the strain, we displayed bADA2 on the surface of *E. coli* outer membrane vesicles (OMVs). Further, we investigated OMV-bADA2 particles as a potential delivery tool for localized adenosine depletion in the tumor microenvironment. We used the well-established Lpp’OmpA anchoring domain for surface display of bADA2 [43]. Meanwhile, the naïve OmpA was retained to avoid potential membrane destabilization [50].

OMVs have previously been used for enzyme display and packaging, and have shown promising potential for maintaining enzymatic activity and stability [51]. For example, Park and colleagues engineered OMVs to display a multienzyme cascade for cellulose hydrolysis. Their results showed significantly higher glucose production than free enzymes, which supports the potential of OMVs as stable and functional biocatalytic nanoparticles [52]. In Su et al. report, a similar strategy was used to functionalize OMV surfaces for enhanced pesticide degradation. They engineered OMVs by recombinant expression of a fusion construct containing organophosphorus hydrolase (OPH) and a bacterial membrane-anchoring partner, ice nucleation protein (INP), in *E. coli*. Additionally, they designed a fusion of cellulose-binding domains (CBD) with Lpp’OmpA to display a second protein, which improved OMV recovery and allowed repeated use of the engineered enzyme [53]. Further to immobilization of enzymes on the surface of OMVs by genetic engineering, other investigators, including Alves et al. and Thakur et al., explored the feasibility of packaging enzymes within the OMV lumen. These studies clearly demonstrated that the encapsulated enzymes retained their catalytic activity [50,54]. This is important because even when a surface-displayed enzyme is partially localized within the lumen, it can still retain its functional activity. In a recent study, Gong et al. demonstrated an efficient enzyme-delivery system using engineered *E. coli* Nissle 1917 to produce enzyme-loaded OMVs in situ. These OMVs were able to cross the gut epithelium, enter the circulation, and maintain stable enzymatic activity [55]. These findings highlight a significant feature of enzyme-loaded outer membrane vesicles (OMVs) as a safe platform for systemic delivery of therapeutic enzymes in the near future.

Our results demonstrate that the purified OMV-bADA2 particles exhibit strong adenosine deaminase activity using a routine colorimetric commercial assay. This confirmed effective loading and functionality of the displayed enzyme. We also observed that extending the enzymatic reaction to 20 min further increased the calculated activity, indicating the system stability and sustained catalytic performance. OMV-bADA2 particles were also tested in serum-free medium spiked with adenosine to assess functionality in a more physiological context. The OMVs efficiently depleted adenosine over time, supporting their potential to operate effectively in complex biological environments. The stability of OMV-bADA2–associated adenosine deaminase activity in mouse serum and PBS over 24 h suggests that OMVs preserve enzymatic function, supporting their use as a robust ADA2 delivery platform and potentially enabling extended dosing intervals in vivo. These characteristics, along with the ease of bacterial expression and OMV incorporation, suggest that bADA2 is a promising, scalable candidate for OMV-based applications and warrants further comparative evaluation with existing human ADA enzymes.

We used *E. coli* ClearColi^®^ strain for the generation of OMVs because its naïve OMVs have a much lower capacity to induce inflammation and do not substantially alter the immune cell composition within the tumor microenvironment [56]. This enabled us to more accurately assess the specific immunomodulatory effects of ADA2, without interference from strong innate immune responses that conventional *E. coli* OMVs can trigger [57]. We administered OMVs via intratumoral injection, which allowed for localized delivery and reduced systemic immune exposure. However, repeated systemic administration may increase the risk of anti-OMV antibody induction and accelerated blood clearance, which prioritizes careful evaluation of these effects in the next stage of preclinical development for our OMV-bADA2 platform.

In both our flow cytometry and immunohistochemistry experiments, we observed a significant increase in CD8α^+^ T cell populations in the OMV-bADA2 treatment groups. Additionally, in our subcutaneous KPC model, intratumoral injection of OMV-bADA2 significantly reduced collagen within the tumor stroma, indicating attenuation of the desmoplastic response. OMV-bADA2 was administered intratumorally at 0.5 and 5 µg per injection, corresponding to 4.8 × 10^7^ and 4.8 × 10^8^ particles per dose, which is consistent with the ~1–10 µg/mouse range reported for bacterial extracellular vesicles and with prior intratumoral OMV dosing regimens [58]. Previous studies across diverse tissues and experimental models, including lung fibrosis in ADA-deficient mice, have demonstrated that adenosine signaling through A2A and A2B receptors directly promotes collagen synthesis and fibrosis, whereas genetic or pharmacologic disruption of this pathway inhibits collagen deposition [11,59,60,61,62].

α-SMA staining was also reduced in OMV-bADA2–treated tumors compared with controls. This marker is widely used as a reliable indicator of activated myofibroblasts, including myofibroblastic cancer-associated fibroblasts (myCAFs) within the tumor stroma [63,64]. In PDAC, myCAFs with high α-SMA expression constitute a major stromal subpopulation that drives extracellular matrix deposition and desmoplastic tumor stroma. These α-SMA-high myCAFs are located in close proximity to tumor cells and account for nearly 50% of all CAFs, and thus represent the dominant CAF subset within the human PDAC stroma [65]. In this sense, the observed reduction in α-SMA staining following OMV-bADA2 treatment suggests a decrease in the activated myCAF pool and a relaxation of the desmoplastic stroma.

In conclusion, here, we presented a novel, efficient, and scalable approach for enzymatic modulation of the TME using engineered bacterial OMVs. Our findings offer a strong rationale for advancing OMV-based ADA delivery as a next-generation immunotherapy platform targeting adenosine-driven immune evasion in PDAC and potentially other solid tumors. Given the versatility of OMVs, further modification of these particles, such as the incorporation of chemotherapeutic agents or additional immunotherapies, could enhance their antitumor efficacy.

## Data Availability

The original data presented in this study are included in the article. Further inquiries can be directed to the corresponding authors.

## Supplementary Materials

The following supporting information can be downloaded at https://www.mdpi.com/article/10.3390/pharmaceutics18080920/s1, Figure S1: SDS-PAGE and Coomassie staining of *E coli* total lysates, outer membrane fraction, and outer membrane vesicles.
