# Supplementary material for: ADA2-Functionalized OMVs Remodel the Tumor Microenvironment in Pancreatic Cancer
Source: Pharmaceutics. 2026 Jul 27;18(8):920. doi: 10.3390/pharmaceutics18080920 (PMC13516308; doi:10.3390/pharmaceutics18080920)
Supplement: Supplementary file 1 [file pharmaceutics-18-00920-s001.zip › pharmaceutics-4416829-supplementary.pdf]

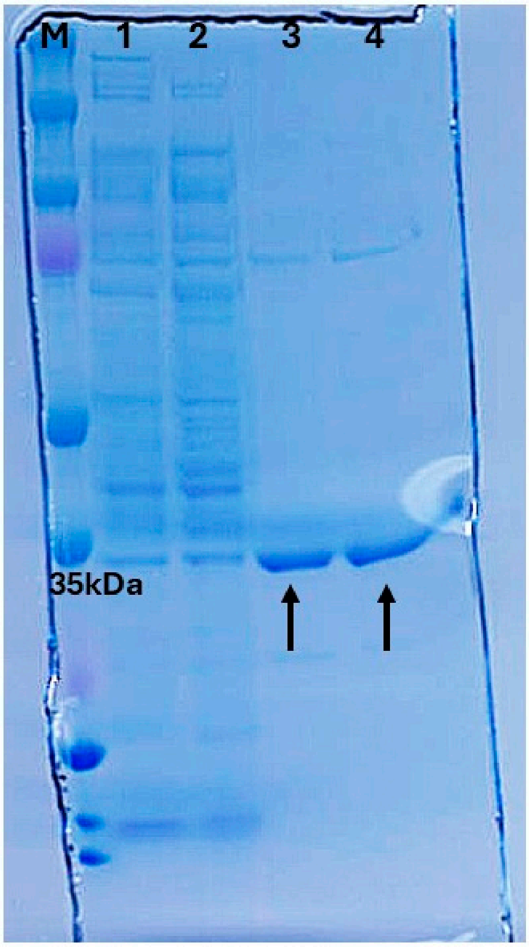

Supplementary Figure S1. SDS-PAGE and Coomassie staining of *E coli* total lysates (lanes 1 and 2), outer membrane fraction (lane 3), and outer membrane vesicles (lane 4). Arrows indicate enriched outer membrane proteins in lanes 3 and 4.
